# Is the molecular microenvironment of the latent HIV reservoir predictable using deep learning approaches?

**DOI:** 10.1128/jvi.00175-26

**Published:** 2026-04-30

**Authors:** Heng-Chang Chen

**Affiliations:** 1 Quantitative Virology Research Group, Population Diagnostics Center, Łukasiewicz Research Network – PORT Polish Center for Technology Development 432080, Wrocław, Poland; Universiteit Gent, Merelbeke, Belgium

**Keywords:** human immunodeficiency virus, molecular microenvironment of the latent HIV reservoir, HIV latent reservoir, deep learning, HIV integration sites, HIV transcription

## Abstract

Although current antiretroviral therapy effectively suppresses viral replication in infected individuals, infections remain incurable. One of the main reasons for this is the uncertainty of the molecular microenvironment of the latent HIV reservoir. If this microenvironment can be precisely targeted, the effectiveness of current antiretroviral therapy can be leveraged. To better depict the potent microenvironments of the latent HIV reservoir, it is crucial to first understand the mechanisms that make some viruses transcriptionally active and others silent in the first place. It is currently feasible to predict gene activity from DNA sequence, coupled with epigenetics, using deep learning approaches. Substantial progress has been made in creating multidimensional data sets related to the HIV latent reservoir. The implementation of deep learning to mine potent attributes, enabling the characterization and prediction of the microenvironment of the latent HIV reservoir, is thus imperative. This minireview discusses potent features that can be integrated into deep learning methodologies to predict the likelihood of the potential microenvironment from different tiered levels of the latent HIV reservoir organization, and proposes the principal workflow to tackle this question using deep learning approaches.

## THE HETEROGENEOUS NATURE OF THE LATENT HIV RESERVOIR: THE PRESENCE OF THE MOLECULAR MICROENVIRONMENTS

The heterogeneous nature of the human immunodeficiency virus (HIV) reservoir can be considered from different perspectives, including distinct reservoir cell types and specific anatomical sanctuaries (1–3), the genome integrity of a provirus (intact versus defective provirus), a bias of HIV integration sites (ISs) associated with its transcription and reactivation, and replication fitness. Although each of these proposed mechanisms can already reflect aspects of the presence of the molecular microenvironments (MMEs) in the latent HIV reservoir (hereinafter, latent MMEs), it is presently impossible to precisely target latent MMEs, rendering the ineffectiveness of current antiviral therapy (ART) and functional cures. Advanced computational approaches that enable not only mining of the masked features hidden in an intricate data set but also concatenating information retrieved from different “omics” readouts related to latent MMEs, subsequently strengthening the biological interpretation of multigenomic outputs, hold paramount importance.

At present, it remains challenging to precisely define and measure latent MMEs, in great part due to the lack of standard methods to access them. For this reason, in this minireview, I attempt to discuss potential features associated with different tiered levels of the MME organization, including HIV IS (Section I), reservoir cellular states in the regulation of HIV transcription (Section II), the integrity of the HIV genome (Section III), and insert-specific HIV transcription and latency reversal (Section IV). Furthermore, I offer my perspectives regarding the rationale of the prediction of latent MMEs using *deep learning* (*DL*) approaches (Section V). Lastly, I propose a theoretical workflow, describing critical steps required to perform this task. Current limitations of *DL* models when used for prediction and my perspectives on how to enhance the explainability of prediction results on latent MMEs based on small-sized HIV cohorts are also provided. To facilitate reading for experimental virologists, I offer a glossary related to *DL* modeling (which includes all terms written in *italics*) at the end of this article.

### Section I—potential features related to HIV IS

HIV DNA integration into the host genome is one of the characteristics in the life cycle and is critical for a persistent infection in reservoir cells. The selection of an HIV IS is, by nature, not a random process throughout a linear genome and has been investigated for a long time (4–6). At a genomic level, HIV ISs are preferentially present in actively transcribed genes when cell lines are infected with HIV *in vitro* without the selection for a latent phenotype (4, 7). Other preferences of HIV IS observed in the inducible J-Lat cellular clones include alphoid repeats in the centromere, gene deserts, and highly expressed genes compared to the constitutively expressed clones (5).

At the level of the 3D genome organization, a bias of HIV IS was observed to correspond to loci in physical proximity to the nuclear pores (8) and active chromatin regions (A1 and A2 compartments) associated with super-enhancers overlain with chromatic immunoprecipitation sequencing (ChIP-seq) signals of the acetylation of lysine 27 on histone H3 (H3K27ac) and trimethylation of lysine 36 on histone H3 (H3K36me3) in a 3D genome (9). In addition, topological signatures, including topologically associating domain boundaries and CCCTC-binding factor (CTCF) (2) and R-loops (DNA:RNA hybrids) (10), also coordinate with HIV IS. HIV integration can be directed by RNA splicing helicase Aquarius—a HIV integrase and LEDGF/p75 binding partner—into R-loop regions positioned in intronic sequences of prevalently transcriptionally active genes, whereas its depletion impairs severely overall integration and retards the remaining integrations out of R-loops and toward heterochromatin-enriched regions (10). Given that R-loops are critical for gene expression (both sense and antisense transcripts [ASTs]) (11, 12), a further investigation of R-loop-dependent mechanisms in terms of HIV sense and antisense transcript initiation and RNA polymerase II pausing on both HIV long terminal repeats (LTRs) is imperative to gain more insights into the mechanism by which HIV ISs dictate its insert-specific transcription (see Section IV). It is important to note that although a similar spatial pattern of HIV IS bias in microglia cells—HIV brain reservoir cells—was observed, the discrepancy exists (2). These findings may suggest that cell-type-specific features should be specified for classification performance in the future, even though it currently remains arduous to achieve substantial accuracy of prediction using *DL* models in the cell-type-specific context (13).

### Section II—potential features related to reservoir cellular states in the regulation of HIV transcription

HIV latency is largely accepted as a consequence of viral tropism for activated CD4+ T cells, which can transition to a resting memory state that is non-permissive for replication (14), indicating that, to some degree, provirus latency resulting from HIV transcription can be controlled by the host-cell activation state since relaxation of activated lymphocytes to a resting-memory state (15, 16). This transition allows integration of the proviral genome in a host cell lacking favorable conditions for optimal viral gene expression, thereby escaping rapid destruction of the infected T cell (17, 18). Latent HIV proviruses can persist in resting memory CD4+ T cells, known as latent reservoirs, with an estimated half-life of 44 months (17–19); treatment is thus lifelong. Although it is still debated regarding the role of naïve T cells in HIV persistence, memory T-cell subsets, including central memory (T_CM_), effector memory cells (T_EM_), and transitional memory (T_TM_), which serve as the main reservoir cells harboring latent infections (20–22), are definite. A recent study observed the inducible reservoir shifts toward a more differentiated T-cell compartment over time after increased time on ART, based on the acute and chronic cohort, suggesting the dynamics of the composition of reservoir cells alongside HIV infection on ART (23). In addition, it is worth noting that a resting-memory state of the reservoir cells has been reported to be correlated with increased epigenetic silencing of the HIV promoter and increased cytoplasmic sequestration of transcription factors that activate HIV transcription (24, 25). The influence of the epigenetic regulation on HIV transcription will be detailed in Section IV.

It is, however, important to stress that, in physiological infection conditions, the latent HIV reservoir can be established early during the acute phase of the infection, possibly before the virus appears in the systemic circulation (23, 26), resonating with a theory that suggests that HIV has evolved a mechanism for rapid establishment of latent infection to facilitate transmission across mucosal barriers (27, 28). Latency was therefore proposed to serve as a “bet-hedging strategy” that allows some infected cells to survive long enough to transit the mucosa. Another more recent study identified a subset of latent cells associated with distinct features from a pool of infected cells with latent infections (29). Remarkably, HIV ISs in this subset of latent cells were prone to be in non-genic regions and in proximity to zinc-finger genes and heterochromatin regions (29). Such biases of integration resemble the features used for the characterization of the reservoir harboring intact proviruses in the status of deep latency in elite controllers (30) or individuals with HIV under prolonged ART, resulting from host immune selection (31) (see Section III). Nevertheless, a continuation of interrogation of potential phenotypic signatures, which tag unique populations of the reservoir cells, such as memory T-cell subsets, a tiny fraction of CD4+ T cells that express CD32a and harbor replication-competent proviruses (32), reservoir cells harboring genome-intact proviruses, and the large HIV-infected clonal population (33), spontaneously active HIV reservoirs (34) (see Section III), reservoir cells harboring proviruses at a deeper level of latency (i.e., elite controllers; see Section III), and a subset of latent cells (29) mentioned above will enrich the complexity of features associated with distinct latent MMEs at a cellular level.

### Section III—potential features related to the integrity of the HIV genome

After HIV DNA integration, only 2%–10% of the proviruses remain genetically intact (35–37); others that are genetically defective harbor large deletions, sequence inversions, hypermutations, and defective splice donor and acceptor sites that prevent viral replication (36, 37). At present, reservoir cells harboring intact proviruses are believed to serve as the main sources of viral rebound. Although the role of defective proviruses remains elusive, the study has shown the involvement of defective proviruses in HIV-specific immunity and innate sensing, rather than simply viral genome ‘‘junk” (38). A recent study pointed out the presence of spontaneously active HIV reservoirs, which are dominated by defective proviruses and anticipate HIV-specific immunity in a majority of ART-suppressed people living with HIV (PLWH) (34). A series of studies, based on longitudinal cohort studies of long-term ART-treated individuals and elite controllers—a subset of HIV-infected individuals distinguished by their ability to maintain a state of apparently durable control of HIV replication without the need for antiviral therapy (39, 40)—further introduces that the diverse strengths of immune-mediated selection forces can reshape the configuration of the latent HIV reservoir harboring genetically intact proviruses alongside the acquired immunodeficiency syndrome (AIDS) disease progression (30, 31), refreshing the concept of HIV deep latency (or silencing).

The concept of HIV deep latency originated from the 1990s onward and was updated progressively. A deeper level of HIV latency is most likely referred to as the latent proviruses that are refractory to reactivation, already discriminating from inducible latency and supporting the presence of distinct latent MMEs. These proviruses are often associated with either epigenetic features (e.g., DNA methylation; see Section IV) or the specific chromosomal conformation (e.g., heterochromatin). These characteristics associated with HIV deep latency were unveiled by HIV IS retrieved from elite controllers with a preference for centromeric satellite DNA or in Krüppel-associated box domain-containing zinc finger (KZNF) genes (30). A similar tendency was recapitulated in individuals with HIV under prolonged ART (31, 41). In fact, HIV ISs in heterochromatin regions are already detected in early-treated individuals after 1 year on ART and are progressively enriched after 5 years on ART based on a longitudinal analysis using the acute and chronic cohort (23).

Moving forward, the observations of distinct graph isomorphism based on the Pearson distance between graph networks constructed by HIV-targeted genes retrieved between ART-treated patients and elite controllers using graph-theoretically based tools and the diverse graph evolutionary trajectories simulated by the Markov chain Monte Carlo (MCMC) method also suggest the different intrinsic properties of HIV reservoirs lying between these two types of HIV-infected individuals (42). A recent study further identified the distinct dynamics of longitudinal biomarkers and pathways related to elite controllers and viremic controllers (i.e., HIV-RNA between 50 and 10,000 c/mL): relative to elite controllers, a higher level of CRTAM, LY9, and CD6 and a lower level of VAT1 were detected in viremic controllers (43). All these recent findings strengthen the probability of the existence of distinct latent MMEs at the global level of network organization (42, 44) and the distinct textural nature of latent MMEs harboring proviruses at a deeper level of latency (43). It is worth noting that, at present, the mechanism of elite control (or the mechanism that can push proviruses to a deeper level of latency) remains unclear. The over-representation of the HLA-B*57 alleles cannot cover every elite controller (45, 46), and many of them can still be viremic and develop progressive disease (47), inferring the presence of additional mechanisms, most likely resulting from the host genome standing behind the phenotype of elite control. A more profound identification of biomarkers and genomic features that enable isolation of such unique MMEs from elite controllers will not only continuously reinforce the concept of HIV deep latency but also advance the development of a novel functional cure under the framework of the “block-and-lock” strategy (48).

### Section IV—potential features related to insert-specific HIV transcription and latency reversal

The pioneer studies from Jordan and colleagues have opened a new avenue that HIV transcription is dependent on HIV IS (49). This finding observed in the experimental setting of transformed cell lines using the HIV-based reporter proviruses somehow resembles the classic biological phenomenon, known as Position Effect Variegation (50), stating that the genomic context has an influence on the expression of endogenous and foreign genes in general. The fact that HIV ISs dictate *in vitro* HIV gene expression has been confirmed by a series of studies (5, 51–55), showcasing the epigenetic modifications appearing at the local genomic context surrounding HIV IS play an important role in determining a provirus fate at a transcription level.

#### Impacts of histone modifications on the local genomic context surrounding HIV IS

**Fig 1:**
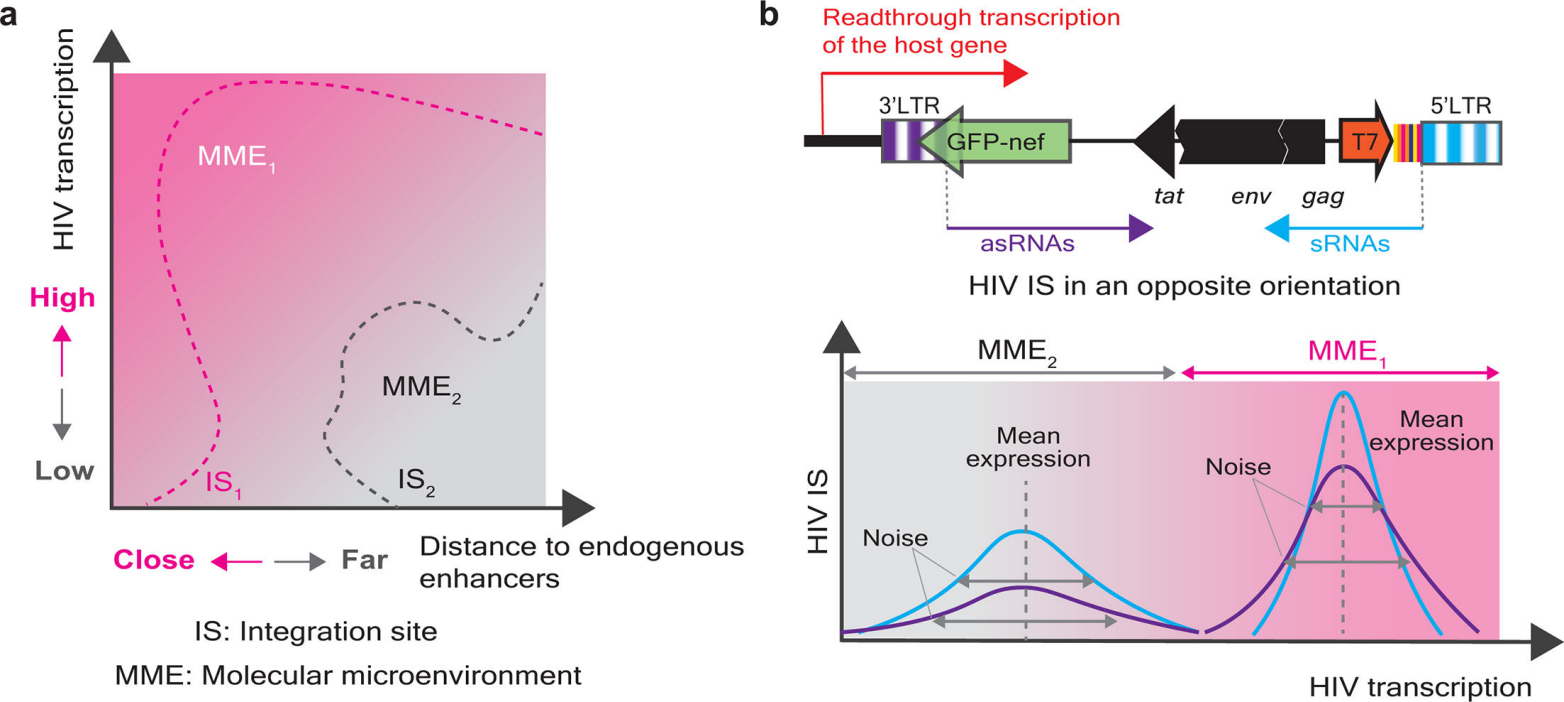
HIV ISs associated with genomic features dictate HIV transcription. (**a**) Heuristic representation of HIV transcription (y-axis) and the distance of an HIV IS to the closest endogenous enhancers, denoted by H3K27ac. An MME characterized by a linear correlation between an HIV IS and a host regulatory element (i.e., an endogenous enhancer) determines the transcriptional phenotype of a provirus. For example, IS_1_ represents a provirus that integrates in the vicinity of an endogenous enhancer, defining the property of MME_1_, which is favorable for HIV transcription. In contrast, IS_2_ represents a provirus that integrates distal to an endogenous enhancer, defining the property of MME_2_, which is unfavorable for HIV transcription. Schematic representation is inspired by Palma, P. (69) https://BioRender.com/4la57ft. (**b**) Holistic representation of HIV transcription resulting from the competition between HIV sense and antisense transcripts versus MMEs. The observation that more than 60% of HIV IS retrieved from elite controllers and long-term ART-treated patients, who displayed an opposite orientation relative to the host gene, leads us to hypothesize that readthrough transcription of the host gene could facilitate HIV antisense transcription, subsequently suppressing HIV sense transcription and promoting the establishment of latent MMEs harboring proviruses at a deeper level of latency. Proviruses that integrate in MME_1_ demonstrate a higher level of transcription and modest transcriptional bursting (i.e., noise). In contrast, proviruses that integrate in MME_2_ demonstrate a modest level of transcription and intense transcriptional bursting. Blue lines illustrate sense RNA transcription; purple lines illustrate antisense RNA transcription; and red lines illustrate readthrough transcription of a host gene targeted by HIV.

A correlation between the HIV IS and the distance of HIV IS to the closest epigenetic modification has been observed (52, 55): the expression of HIV is strongly close to endogenous enhancers denoted by ChIP-seq signals (fold change over control) of H3K27ac (Fig. 1a) in the setting of Jurkat T cells. HIV ISs also influence the response of a latent provirus to latency-reversing agents (LRAs) and latency-promoting agents (LPAs). In the former treatment with LRAs, latent proviruses that integrate in the proximity to endogenous enhancer denoted by H3K27ac are more sensitive to vorinostat compared to latent proviruses subjected to phytohemagglutinin (52). Battivelli and colleagues further estimated that less than 5% of the primary CD4+ T cells with latent infections are inducible by tested LRAs, including JQ1 (56–60), panobinostat (61), and bryostatin-1 (62, 63), and observed distinct epigenetic profiles in terms of H3K27ac, monomethylation of lysine 4 on histone H3 (H3K4me1, active enhancers), trimethylation of lysine 36 on histone H3 (H3K36me3, actively transcribed gene body), trimethylation of lysine 9 on histone H3 (H3K9m3) and trimethylation of lysine 27 on histone H3 (H3K27m3) (64, 65) (both are repressive marks of transcription) associated with HIV IS retrieved from productively infected cells (PIC), reactivated latently infected cells (RLIC), and non-reactivated latently infected cells (NRLIC) (53). Intriguingly, different promoter activities from both HIV LTRs were detected when latent proviruses were subjected to different LRAs (55). In the latter treatment with LPAs, in the *in vitro* infection setting, although infected cells subjected to LEDGINs (66)—small-molecule inhibitors of the interaction between HIV integrase and the cellular factor lens epithelium-derived growth factor/p75 (LEDGF/p75)—enable retargeting of an HIV IS toward genomic regions that are not favorable for HIV gene expression and reactivation (67), a small fraction of retargeted proviruses remains transcriptionally active (68). This observation already suggests that perhaps a precise prediction of latent MMEs cannot be achieved based on merely HIV IS coupled with epigenetic features. Altogether, these findings pave the way for a proposition for reactivation therapies and functional cures that cocktails of drugs with complementary spectra should be developed to cover latent proviruses with different propensities toward drugs across different MMEs. In addition, systematic classification of insert-specific HIV gene expression might be a clue to lead us to the identification of distinct latent MMEs, in which proviruses demonstrate different transcriptional phenotypes even though robust models for the prediction of HIV transcription are presently unavailable (see Section V).

#### Impacts of epigenetic regulation of CpG methylation

Epigenetics comprises several molecules and mechanisms that alter gene expression during a long-term period in the context of the same DNA sequence (70). These mechanisms include DNA methylation and the covalent addition of a methyl group to the fifth carbon of cytosine (71). Epigenetic modulation is extensively involved in the establishment and maintenance of HIV latency. HIV 5′LTR is occupied by two precisely positioned nucleosomes, Nuc-0 and Nuc-1, whose remodeling and modifications directly impact HIV latency (72). Their positioning is tightly bound to various histone modifications (73–77). In addition, the HIV promoter region also contains two conserved CpG islands that form DNA methylation sites in the latent state, which are modified by DNA methyltransferase 1 (DNMT1) and maintained by methyl-CpG-binding domain protein 2 (MBD2) (78–80). They can induce significant changes in the host epigenome, leading to a cellular environment more favorable to its replication and persistence (81). Accumulating studies have reported that HIV infection leads to an increment in the average genome methylation status—both hypermethylation and hypomethylation changes are observed in the host genome (82–88), which may serve as potential MMEs, in which HIV IS can be maintained under the pressure of immune-mediated selection (30, 31), consequently resulting in the persistence of proviruses at a deeper level of latency. Lastly, unique *in vitro* cellular models, in which proviruses exhibit phenotypic bifurcation in terms of fluctuations in HIV transcription, strengthen the hypothesis that, in some cases, the regulation of HIV transcription can be a pure epigenetic phenomenon (55, 89).

#### HIV transcription machinery

HIV transcription has a long and rich history of investigations and is well known to be tightly regulated by the 5′LTR promoter (90–92), with which HIV hijacks host cellular transcription factors to control its gene expression (93). In brief, upon infection, the recruited host cell transcription factors are sufficient to initiate HIV transcription; however, very low levels of full-length transcripts are produced at this stage (94, 95). To increase expression, the HIV transactivator Tat protein plays an important role by enhancing transcriptional elongation from the HIV 5’LTR promoter (96), which is referred to as the promoter ON state. Eventually, the Tat positive-feedback circuit relaxes in the OFF state, thus leading to the establishment of latency (27, 89). At present, the two-state promoter model (ON *versus* OFF) represents the classic model for HIV transcriptional noise, an episodic process of gene expression (97, 98), and has been applied to explain the molecular mechanism behind this bursty phenotype of gene expression. This two-state model relies on a specific regulatory architecture to determine thresholds that allow a biological system to either respond or disregard an input signal. The HIV Tat transactivation is sufficient to induce a burst of HIV 5′LTR promoter activity that leads to fluctuations in HIV gene expression (27); however, this model fails to generate a clear threshold response with deterministic bistability. In addition, this model fails to represent the physiological HIV infection condition that HIV latent infection can be established in the early stage of infection, as mentioned in Section II. All these observations imply that most likely an additional unknown determinant or mechanism is involved in governing stochastic HIV transcription. Mathematical modeling recapitulating the regulation of HIV transcription can be referred to (69, 99). Most likely, the specificity (e.g., incorporation of more parameters) of these algorithms will be necessary for a better representation of distinct latent MMEs when additional features related to unique latent MMEs are identified.

#### Potential role of ASTs in promoting latency

Viruses have developed various strategies to establish a latent state to ensure their persistence in their hosts. For instance, herpes viruses encode latency proteins that are responsible for switching the viral cycle from a lytic to a latent state (100). Another example is the detection of antisense transcripts, namely CMV latency-associated transcripts in bone marrow aspirating from naturally infected, healthy seropositive donors infected by human cytomegalovirus (101). Retroviruses, in contrast, are known to persist through transcriptional silencing and do not encode latency proteins. In fact, in other human retroviruses, like human T-cell leukemia virus type 1 (HTLV-1), one of the best characterized ASTs in a viral system, its antisense gene product, HTLV-1 basic leucine zipper factor (HBZ), has been proven to functionally promote the survival and proliferation of HTLV-1-infected cells, thereby influencing progression into adult T-cell leukemia/lymphoma or HTLV-1-associated myelopathy/tropical spastic paraparesis (102). HIV ASTs are supposed to suppress sense RNA transcription (103–105); however, at present, the fundamental mechanism behind the competition between sense and antisense transcripts and whether or not HIV ASTs can actively promote the establishment of HIV latency remains unclear.

Our understanding regarding HIV antisense transcription, which was first postulated in 1988 (106), remains insufficiently explored. Multiple HIV ASTs with both protein-coding (107) and non-coding functions (103–105, 108) are driven by the Tat-independent HIV 3′LTR (109–111) and compete with its sense RNA transcription (55). The presence of ASTs in unstimulated peripheral blood mononuclear cells collected from PLWH, along with the ectopic expression of ASTs that suppress HIV transcription and latency reversal (112), further suggests their natural occurrence in PLWH (113). Intriguingly, one preprint suggests that the expression of HIV ASTs is associated with the efficiency of HIV IS (114); however, further investigation is required. It is important to stress that more than 60% of HIV IS retrieved from elite controllers and long-term ART-treated patients, who displayed an opposite orientation relative to the host gene. This observation may infer that readthrough transcription of the host gene could facilitate HIV antisense transcription, subsequently suppressing HIV sense transcription and promoting the establishment of latent MMEs harboring proviruses at a deeper level of latency (Fig. 1b). In contrast, transcriptional interference occurs between a host gene readthrough transcript and HIV sense transcript (Fig. 1b), subsequently leading to the establishment of HIV latency. Nevertheless, our empirical observation also indicates that, most likely, features at the RNA level alone may be too strict (or insufficient) for the classification of stochastic HIV transcription (55).

#### Latency reversal and spread between cells

Current antiretroviral therapy can block new infections of susceptible cells, but cannot eliminate the virus production from cells with integrated latent proviruses. Latently infected cells that form latent reservoirs may cause viral rebound after antiretroviral therapy is interrupted, consequently impeding the efficacy of treatment. Ongoing natural reactivation of latently infected cells results in virus release in lymphoid tissue (115) with a low viremia on the order of two HIV RNA copies/mL of plasma (116, 117). Upon discontinuation of ART, exponential HIV growth rebounds within a few weeks (118, 119), aligning with a cascade of stochastic processes (120). An accurate prediction of the behavior of provirus rebound is therefore crucial for the better design of antiretroviral regimens. Ordinary differential equations, which were used to model kinetics, were applied to model HIV rebound following ART interruption more than 20 years ago (118, 119). Stochastic modeling, such as MCMC algorithms (121, 122), the Gillespie algorithm (123), and Bayesian inference (124), is also implemented in forecasting viral rebound after ART is interrupted (125, 126). A variety of predictive variables were utilized to establish models, including the decay rate of the latent reservoir in the absence of viral replication, the rate at which the latent reservoir produces actively infected cells, the probability that any one activated cell will produce a rebounding infection before its lineage dies, the net growth rate of the infection once restarted, the numbers of resting memory and latently infected CD4+ T cells as well as HIV RNA copies (125, 126). At present, the parameter related to the variety across patients is not yet frequently implemented in the model construction. A recent study, however, points out that clinical characteristics, for example, sex, influence the composition of the HIV reservoir associated with immunity (127). In the future, parameters at the population and cohort levels should also be included when predicting latent MMEs associated with latency reversal.

### Section V—prediction of latent MMEs using *DL* approaches

**Fig 2:**
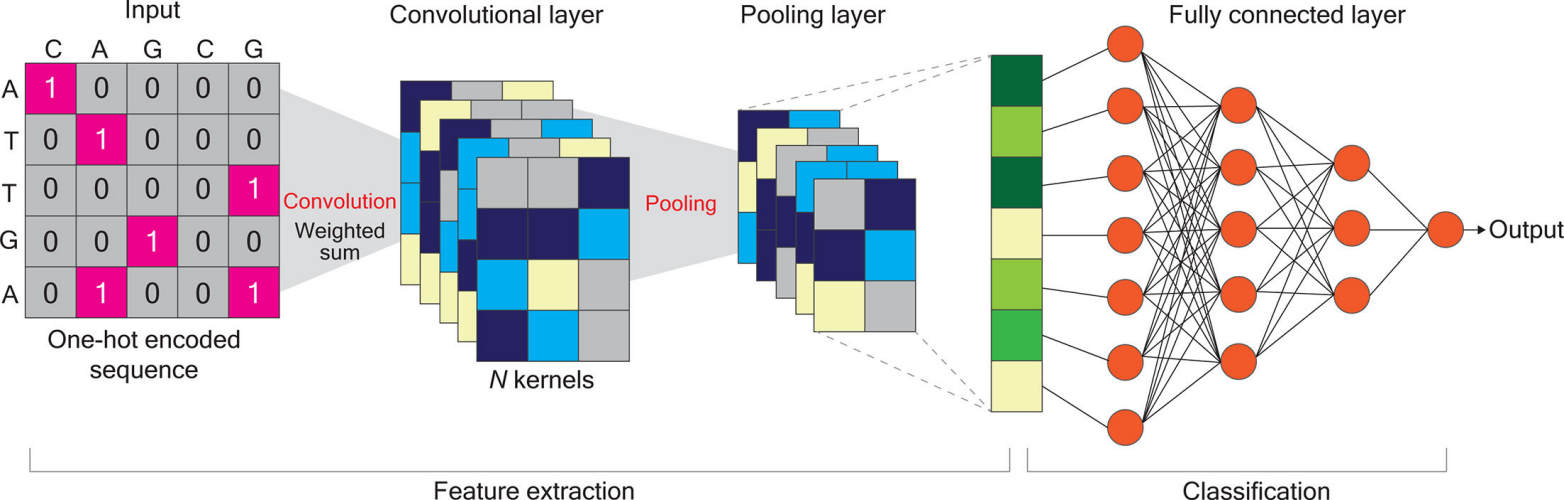
Architecture of the CNNs. CNNs are feed-forward neural networks that are used for image classification and processing with the help of provided data. A hierarchical architecture in CNNs implements the initial layer that captures local and simple features from input data sets, and the deeper layers, including the convolution, pooling, and fully connected layers, that can dissect more intricate interactions across entities. The convolution layer is the core component in CNNs. See Box 1 for details.

**Fig 3:**
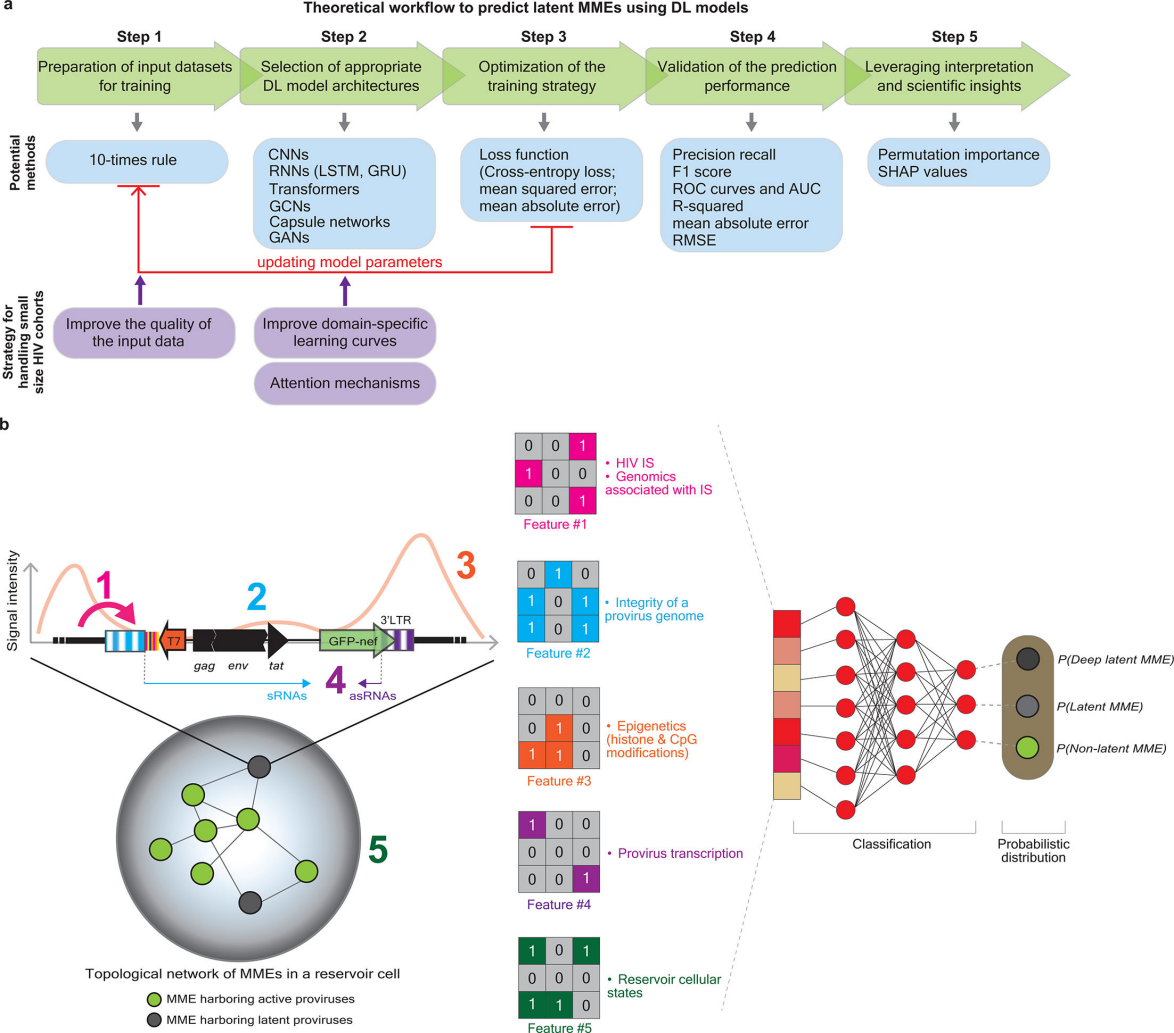
Schematic representation of a theoretical workflow and the rationale of the construction of a DL model for the prediction of the likelihood of latent MMEs. (**a**) A theoretical workflow (highlighted in green boxes), consisting of the five steps, is proposed to predict the likelihood of latent MMEs using DL models. Available tools that can be used for each step are provided in blue boxes. Potential solutions to handle a small size of an HIV cohort for modeling are provided in purple boxes. CNNs, convolutional neural networks; RNNs, recurrent neural networks; LSTM, long short-term memory; GRU, gated recurrent unit; GCNs, graph convolutional networks; GANs, generative adversarial networks; RMSE, root mean squared error; SHAP values, SHapley Additive exPlanations values. (**b**) Multi-omics-based features obtained from various biological dimensions will be used as input, followed by the convolution, pooling, and fully connected layers, enabling a superior performance in predicting the likelihood of latent MMEs. Five groups of features appearing at different levels of the MME organization are discussed in the minireview. Please refer to the main text for details.

A low frequency of proviruses (0.01%–0.1%) in CD4+ T cells (128) renders it difficult to precisely target latent MMEs, subsequently impairing the effectiveness of current ART. The success of an accurate prediction of latent MMEs relies not only on the understanding of the mechanisms that determine the fate of a provirus but also on a powerful tool that enables the analysis of a massive amount of sequence data covering the nature of latent MMEs from numerous tiers. *DL*, a subdiscipline of machine learning (ML), yielding *end-to-end models* (e.g., *deep neural networks*) to compute more complex features, is deemed to be a potential option. Several *DL* models and algorithms have been available, and the field is constantly evolving. In addition to fundamental models, such as *convolutional neural networks* (*CNNs*) (see Box 1; Fig. 2) and *recurrent neural networks* (*RNNs*) (129), recent innovations in *DL* architectures include *transformers* (130, 131), *graph convolutional networks* (*GCNs*) (132), *capsule networks* (133), *generative adversarial networks (GANs*) (134), and advances made in training techniques. Due to the limitation of the text length, a brief description of each model is provided in a glossary. The principal components embedded in a *DL* model are described in Box 1, taking *CCNs* as an example. In the following content, the emphasis will be placed on the discussion of the rationale and the potential workflow (Fig. 3a) for the examination and prediction of the likelihood of latent MMEs.

Box 1. **Architecture of convolutional neural networks**

The design of the model’s architecture, which is referred to as the structure of the layered network, including the types of operations performed in each layer, the number of layers, and how they are connected, is crucial to achieving accurate predictions (135). A hierarchical architecture in *CNNs* implements the initial layer that captures local and simple features from input data sets, whereas the deeper layers (i.e., *the convolution, pooling,* and *fully connected layers*) dissect more intricate interactions across entities (135) (Fig. 2). The convolution layer is the core component in *CNNs* (136, 137). This layer performs a dot product between a filter, namely, *kernel* (Fig. 2), and a subset of the sequence matching the *kernel* size. Each convolution operation corresponds to a *position weight matrix*. Given that this model architecture can be regarded as a series of arithmetic operations applied to its input, it is imperative to represent inputs, such as DNA sequences, in a numerical format (i.e., quantitative scores) for quantitative modeling. Most commonly, *one-hot encoding* is used, in which each nucleotide in a sequence profile is converted to a unique binary vector of length 4, with each dimension corresponding to a nucleotide type. For example, A = [1, 0, 0, 0], T = [0, 1, 0, 0], C = [0, 0, 1, 0], and G = [0, 0, 0, 1].

The output of a neuron after the convolution operation can be described by equation 1.

(1) $${output}_{j}=act\left(\sum_{t=1}^{n}{\left(W*X\right)}_{tj}+bias_{l}\right)$$

The variable act is a chosen activation function, *W* is the weight matrix or *kernel*, *X* is the input vector, * is the convolution operator, and *n* is the total number of nodes that the neuron is receiving input from.

After the convolution operation, a pooling operation may follow in a *pooling layer* (138). The common pooling operations are max pooling and average pooling. Afterward, the fully connected layer follows *the pooling layer* before output is generated (Fig. 2). Rather than choosing features manually or in a preprocessing step, *CNNs* adaptively learn them from the data during training and apply nonlinear transformations to map input data to informative high-dimensional representations that trivialize classification or regression (139).

#### Use of the DL model to predict HIV IS

*DL* models have been frequently applied in healthcare. Application domains include medical imaging, genomics, and drug discovery, patient monitoring and care, and federated learning and privacy. With respect to the HIV field, *DL* models have been utilized for diagnostics, drug resistance prediction, neuroimaging, comorbidity detection, and the prediction of HIV IS (140–142). DeepHINT (Deep learning for HIV integration), which is an *attention-based DL* framework, enables the prediction of an HIV IS and mechanistic explanations (i.e., sequence motifs) of the detected sites based on either primary DNA sequences alone or together with the data set of ChIP-seq H3K36me3 signals (140). *Attention mechanisms* are particularly designed in this framework to extract important regions of input data by training an additional neural network, subsequently increasing the explainability of *DL* models. In principle, DeepHINT first employs multiple convolution-pooling modules to automatically learn informative sequence features from the DNA sequence 1 kilobase (kb) upstream and downstream from the HIV IS, followed by *one-hot encoding*, the operation of a *position weight matrix*, and the incorporation of the *attention mechanism* (140). Altogether, DNA sequences as input alone enable the prediction of an HIV IS; the addition of the H3K36me3 signal as an input leverages prediction performance (140). The feasibility of predicting HIV IS merely based on the DNA sequence surrounding HIV IS already sheds light on the possibility of predicting the likelihood of latent MMEs if HIV gene expression can also be accurately forecasted. The critical question now is whether HIV transcription (or gene expression in general) is predictable using *DL* models as well.

#### Rationale behind the application of DL models to predict the likelihood of latent MMEs

Even though current research employing *DL* models for the prediction of HIV latency is scarce—of note, three research articles were released using NCBI PubMed with the keywords ((HIV latency) OR (HIV latent reservoir)) AND ((deep learning) OR (deep neural networks) OR (Convolutional Neural Networks)), accessed on 6 March 2026*—DL* has been progressively implemented in HIV studies in the last years. In addition to the prediction of HIV IS (140), based on HIV sequence data, Steiner and colleagues applied a *multilayer perceptron* (*MLP*) (143), a *bidirectional recurrent neural network* (*BRNN*) (144), and *CNNs* for drug resistance prediction (145). Dampier and colleagues applied HIV-Bidirectional Encoder Representations from Transformers (BERT) (146)—a protein-based *transformer* model—to predict protease drug resistance, the coreceptor tropism based on the V3 loop sequence, and the presence of HIV reservoirs across anatomical sites (147). Derbel and colleagues established a deep learning framework (148), namely Rep2Mut, which is the evolutionary scale modeling protein language model, to classify the difference between wild-type HIV Tat protein sequences and mutated sequences (149). Arrigoni and colleagues applied *variational autoencoder* (*VAE*) (150)-based models to screen potential new ligands of the enzyme, which do not belong to any known class of HIV protease inhibitors (151). Powell and Davis extended the highly expressive cryoDRGN *DL* architecture (152, 153), namely tomoDRGN (Deep Reconstructing Generative Networks), to reveal the high-level organization of HIV capsid complexes (154). Kutsal and colleagues applied the tailored *long short-term memory* (*LSTM*) (155) *VAE* architecture to the discovery of new antiretroviral drugs (156). Pham and colleagues developed DeepARV, a *transformer*-based *DL* model, to predict drug-drug interactions in PLWH subjected to different antiretroviral drugs and a wide range of comedications (157).

In fact, DNA-barcode-based high-throughput technology has evidenced the effects of chromatin position on gene expression (158), aligning with the dogma of position effects (159) (see Section IV). In addition, the genome-wide mapping of autonomous promoter activity demonstrated that the transcriptional activities associated with isolated promoters can explain a significant amount (54%) of endogenous promoter activity (160). These results already established a clear mechanistic link between the primary DNA sequence of promoters and variability in gene expression levels. Since then, these massively parallel reporter assays (MPRAs) have made substantial progress (135) and provided an independent source of training data by directly measuring the ability of thousands to billions of short DNA sequences (up to ~2 kb) (161–163). Such a so-called *sequence-to-expression* (*S2E*) *model* exemplifies the capability of extrapolating previously unseen sequences and predicting the effect of sequence variants or generating entirely synthetic regulatory elements not present in the genome (135). Since HIV transcriptional activity is largely influenced by HIV IS and the local genomic environment, the *S2E models* may offer a promising approach to decipher the language of DNA sequences associated with HIV IS and efficiently predict HIV transcription and distinct MMEs.

In addition to its capacity in sequence-level prediction, *DL* models enable the capture of non-linear relationships between predictive variables and HIV transcription, resulting from, for example, bifurcation of proviral fate and stochastic HIV transcription (55, 89, 97, 98). Furthermore, *DL* models overcome the curse of dimensionality (164, 165). Their success has been exemplified in game playing (166), computer vision (167), natural language processing (168), and computational biology (169). This noticeable advance facilitates the integration of single-cell RNA-seq (scRNA-seq) data for prediction, given that the latent reservoir is extremely rare and heterogeneous (128). Methods, such as VASC (170), scVAE (171), scVDN (172), and ScInfoVAE (173), have been developed under the *DL* framework for the analysis of scRNA-seq data. Furthermore, *GCNs,* which can embed millions of single cells into meaningful low-dimensional manifolds (see the following section Step 2), are an additional option for input data at a single-cell scale. Although at present, *DL* models have not yet been systematically tested for their effectiveness in predicting HIV transcription and latent MMEs, the previous study based on binary logistic regression models constructed by 26 regulatory features, including lamin sub-compartment states (denoted by Hi-C), H3K27ac (denoted by ChIP-seq), and chromatin accessibility (denoted by MNase-seq), has showcased the feasibility of using AI-based approaches (174). The modest prediction outcomes (area under the receiver operating characteristics, 64.71%) from this traditional ML may be caused by the intricate nature of HIV transcription and latent MMEs (174). With the aforementioned technical breakthroughs in *DL*, it can be expected that *DL* models will offer superior predictive performance. It is important to stress that even though traditional ML, which is based on feature vectorization of input sequences, can also be applied for sequence-based prediction, *DL* with more advanced tokenization techniques further leverages prediction sensitivity, specificity, and accuracy. For example, Bhandari and colleagues used the frequency-based tokenization approach to enhance *CNN* efficiency in promoter prediction across diverse species (175). Fallahpour and colleagues designed a specialized and tailored tokenization scheme embedded in a *transformer* model to generate codon-optimized DNA sequences (176). Other tokenization approaches on DNA sequences, including nucleotide/protein-based, word-based, *k*-mer-based, and subword tokenization, can be referred to in the review from Testagrose and Boucher (177).

#### Theoretical workflow to predict latent MMEs using DL models

##### Step 1—preparation of input data sets for training

Although *DL* models are known for their high data demands, it continues to be difficult to pinpoint the correct sample size for the specific prediction target in the first place. With this respect, the 10-times rule (178) has been a favorite due to its simplicity of application. This rule means that the number of data sets should be 10 times more than the number of parameters embedded in a data set. Given that HIV research frequently suffers from a small cohort, one potential solution that can mitigate this issue is to leverage the quality of the input data in terms of the specificity of the parameters. For example, features precisely target proviruses at a deeper level of latency or those specific to certain LRAs or LPAs. In addition, a quality control procedure should also be implemented to filter out problematic data and outliers before training. The presently available data sets that can be used to train *DL* models, including (i) HIV IS mapped by a wide range of PCR-based (e.g., ligation-mediated PCR, linear amplification-mediated PCR, Integration Site Loop Amplification [179], INSPIIRED [180], LIS-seq [55, 181]) followed by high-throughput sequencing, (ii) insert-specific single provirus transcriptomics measured by B-HIVE (52, 182), (iii) the integrity of the HIV genome (intact versus defective; studies that characterize the integrity of the HIV genome are summarized in Więcek and Chen [183]), (iv) RNA-seq and host transcriptomics (e.g., available at Gene Expression Omnibus [GEO]), (v) scRNA-seq on cells from PLWH on ART using classic scRNA-seq (184), ECCITEseq (185), DOGMAseq (186), HIV-seq (187), (vi) (epi)genomic data sets (e.g., ChIP-seq, MNase-seq, ATAC-seq, HiC, and so on), (vii) HIV-human proteins interactomics data (e.g., PHISTO [188], NCBI database [189], VirusMentha [190], VirHostNet [191], HPIDB [192], Viruses.STRING [193], and so on), (viii) clinical longitudinal cohort data (longitudinal studies associated with the HIV reservoir are summarized in Chen [44]), and (ix) HIV sequence variation data (e.g., available at the Los Alamos National Laboratory HIV Databases [https://www.hiv.lanl.gov/]).

##### Step 2—selection of appropriate DL model architectures

An appropriate *DL* model can be determined based on the type of inputs, the prediction task, and computational constraints. In principle, *CCNs* are typically suitable for grid-like data sets (see Box 1) (194), whereas *recurrent neural networks* (*RNNs*) (129), particularly *long short-term memory* (*LSTM*) (155) or *gated recurrent unit* (*GRU*) (195) architectures, are optional for sequence-based and time-series data sets. Here, it is worth noting that network-based analysis in “omics” data sets is one of the common approaches to illustrate pathogen-host interactions and has been broadly applied to HIV research (44). With this element in mind, *GCNs* can serve as an option for the prediction of latent MMEs when the input data set is retrieved from scRNA-seq or HIV-host molecular interactomes. Network-embedded *GCNs* have become instrumental in representing complex biological networks in multi-omics integrations. In these models, each entity can be treated as a vertex, and edges unveil statistical similarity or correlation coefficients derived from “omics” data between two adjacent vertices. Unlike traditional *CNNs* (see Box 1), which operate on grid-like data (Fig. 2), *GCNs* learn by aggregating information from neighbors of individual vertices, allowing them to capture contextual signals and relational patterns within graph networks. The core *GCNs* update rule can be described as:


(2) $$H^{\left(l+1\right)}=\sigma (D^{-1/2}\tilde{A}D^{-1/2}H^{\left(l\right)}\Theta^{\left(l\right)})$$

where Ã = *A* + *I* adds self-loops to include information from individual vertices, *D* is the degree matrix, *H*^(^*^l^*^)^ represents vertex features at layer *l*, Θ^(^*^I^*^)^ contains trainable weights, and σ is a non-linear activation function (132). When stacked, multi-layer *GCNs* allow information to flow across the graph, achieving *DL*. *GCNs* exploit both the network topology and feature attributes to classify vertices, enabling interpretable and efficient learning. *Attention-based GCNs* further extend their capacity by adjusting edge weights based on feature relevance, resulting in more robust and interpretable predictions (196). Whether or not *GCNs* and their relative models can facilitate identifying potential graphlets (197, 198)—a set of driver vertices defined as core–peripheral structures and considered to hold paramount importance to underpin the topology of a network—will be a question of interest and requires further investigation. If achievable, these *DL*-based approaches can retrieve the most significant attributes for the classification and prediction of the likelihood of distinct latent MMEs. Overall, suggested *DL* methods for different types of genomic and omics data, as well as strengths and limitations, are summarized in Table 1.

**TABLE 1:** Systematic summary of suggested *DL* models for various input omics data

Input data type	Input data format	*DL* methods	Representative models	Key tasks	Strengths	Limitations
DNA sequence-based	One-hot encoded nucleotide sequences	*CNNs; Transformer* (130, 131); *RNNs* (129); *Long Short-Term Memory* (*LSTM*) (155); Hybrid *CNN-RNN*	DeepSEA (199); DeepBind (200); DNABERT/ DNABERT-2 (201, 202); Nucleotide *transformer* (203)	Transcription factor binding prediction; variant effect prediction; promoter/enhancer identification	Capture of local sequence motifs; modeling long-range genomic dependencies; multi-task learning across regulatory features	High memory usage for long sequences; limited interpretability of learned features; context-specificity issues
Transcriptomics	Gene expression matrices (bulk RNA-seq counts or TPM)	*Autoencoder/VAE* (150); *GANs* (134); *MLP* (143); *DNNs* (137); *GNNs*	scVI (204); ZINB-WaVE (205); DeepLIFT (206)	Gene expression prediction; dimensionality reduction; subtype classification	Handling high-dimensional sparse data; effective for batch correction	Sensitive to batch effects; requires large sample sizes
Long-read sequence	Raw nanopore ionic current signals/PacBio HiFi reads (FASTQ/BAM)	*RNNs* (129); *LSTM* (155); *Transformer* (130, 131); *CNNs; Conditional random fields* (*CRF*) (207) *+ DNNs* (137)	Bonito/Dorado^*a*^; PEPPER-Margin-DeepVariant (208)	Basecalling; structural variant detection; isoform identification; methylation calling	Resolving complex structural variants and full-length transcripts; detection of base modifications simultaneously	Higher raw error rates versus short reads; computationally intensive; requires GPU for real-time basecalling
scRNA-seq	UMI count matrices (cells × genes); sparse format	*VAE* (150)*; Graph transformer; GNNs; Diffusion models* (209)	scVI (204); Geneformer (210); scBERT (211)	Cell clustering and annotation; trajectory and pseudotime inference; batch correction; dropout imputation	Single-cell resolution enables cell-type discovery; foundation models transferable across tissues	Extreme sparsity and dropout noise; the presence of biological variation and technical noise
Epigenetics and genomics	ChIP-seq/ATAC-seq signal tracks; WGBS methylation arrays; Hi-C contact matrices	*CNNs; Transformer* (130, 131)*;* Multi-task *DNNs; Attention-based models*	DeepSEA (199); Enformer (212); DeepMEL (213)	Chromatin accessibility; histone modification prediction; DNA methylation prediction; 3D genome and TAD modeling	Integration of multi-omics signals; decoding regulatory grammar; long-range enhancer-promoter interactions	Context-specific regulatory patterns; class imbalance in peak calling; high data heterogeneity
Network-based	Adjacency matrices (correlation coefficients) and edge lists; biological knowledge graphs; co-expression networks	*GCNs*/*Graph attention networks* (*GAT*) (214); *Graph transformer; Variational graph AE; Message passing NN* (*MPNN*) (215)	DTINet (216); SEAL (217)	Protein-protein interaction prediction; drug-target interaction; gene regulatory network inference; pathway over-representation analysis	Exploiting relational interactions between biological entities; integration of heterogeneous data sources	Incomplete and noisy interaction networks; scalability to whole-genome graphs; over-smoothing in deep *GNNs*
Longitudinal cohort	Time-series omics matrices; EHR^*b*^ records with irregular timestamps; repeated-measures cohort data	*LSTM* (155); *GRU* (195); Temporal *transformer; Neural ODE* (218)	T-LSTM (219); ODE-RNN (220); State space models (Mamba) (221)	Disease progression modeling; time-series gene expression analysis; patient trajectory prediction; microbiome temporal dynamics	Modeling temporal dependencies and dynamic biological processes; handling variable-length sequences	Irregular sampling intervals; missing time points; small longitudinal cohort sizes; temporal batch effects; inter- and intra-patient variations

^*a*^ Bonito/Dorado (ONT basecalling) Oxford Nanopore Technologies (2021–present). Bonito and Dorado: open-source basecallers for Nanopore sequencing https://github.com/nanoporetech/bonito; https://github.com/nanoporetech/dorado.

^*b*^ EHR: electronic health record.

##### Step 3—optimization of the training strategy

Training *DL* models is an iterative process and requires careful monitoring and adjustment. In this step, it is critical to select an appropriate loss function—a mathematical way to measure the quality of a model prediction—that aligns with prediction tasks. Cross-entropy loss (222) is standard for classification, whereas mean squared error and mean absolute error can be applied to regression. In principle, prediction accuracy can be improved by training the model with relevant features of high complexity. It is also important to stress that, in this step, updating model parameters to optimize the algorithm and implementing regularization techniques to prevent overfitting can be carried out.

##### Step 4—validation of the prediction performance

Rigorous evaluation of predictive accuracy, ensuring the experimental quality, is essential, as performed in all artificial intelligence (AI)-based studies. Classical indicators include precision, recall, F1 score, ROC curves, and area under the curve (AUC), *R*-squared, mean absolute error, and root mean squared error. Cross-validation is recommended for a small input data set.

##### Step 5—leveraging interpretation and scientific insights

In addition to completing prediction tasks, it is crucial that *DL* models contribute to extracting interpretable insights from trained models to validate our scientific question: Does the HIV latent reservoir possess distinct MMEs, and are they predictable? The performance of the feature importance analysis, such as permutation importance (223) and SHAP values (224), enabling the quantification of how much predictive variables contribute to prediction, is recommended.

At present, interpretation accuracy using *DL* models is challenging to measure due to the lack of appropriate gold standards (225). The straightforward strategy to improve the interpretability of prediction is the integration of multidimensional data sets for training prediction models (Fig. 3b). It is also worth noting that P-NET (226)—one of the most prominent biology-inspired models trained on patient mutation data—already exemplified the feasibility of preclinical discovery and clinical prediction in prostate cancer using a neural network architecture; the implementation of strengthening control methods in a neural network can still leverage its interpretability (227). In addition, HIV clinical data sets covering the HIV type 1 genomic diversity at the population level and immunity-relevant parameters should also be taken into account. The cohort study based on both partial HIV *pol* gene and viral near full-length genome sequences retrieved from HIV type 1 subtype B demonstrated a heritability of ~10%–20% for the HIV latent reservoir under suppressive ART (228). In parallel, clinical features retrieved from the longitudinal cohort can be taken into account because the dynamics of immunological parameters alongside HIV/AIDS disease progression have been characterized (229, 230). Implementation of longitudinal features may aid in recapitulating the evolution of latent MMEs. Another possible strategy to strengthen the explainability of prediction using *DL* models is the introduction of *attention mechanisms*, which are particularly designed to extract important regions of input data by training an additional neural network that learns the relative importance of each input position from local features (231) and has been implemented in the prediction of HIV IS (140) (see the previous paragraph in Section V).

It is important to note that although multidimensional data sets are critical to achieve better predictive accuracy, how to choose an appropriately sized *CNN* should be prudently examined. This issue can be explicated by the bias-variance dilemma (232)—the trade-off between a model’s ability to accurately recapitulate the underlying data patterns (low bias) and its tolerance to the variations in the training data (high variance). A large neural network may risk overfitting the data, resulting in poor performance. A proposed solution for finding the optimal network size is to continually calculate both the bias and the variance components of the error during training.

## DISCUSSION

The nature of biased HIV IS associated with diverse levels of HIV transcription, allowing us to conceive the presence of distinct latent MMEs. This concept can be supported by clinical and experimental findings showing that DNA sequence, coupled with genomic and epigenetic features, reservoir cell states, and the integrity of the proviral genome, are tightly bound to the regulation of HIV transcription and latency reversal. Although it remains elusive whether the likelihood of distinct latent MMEs can be precisely predicted, the success in the prediction of HIV IS (140), and the accessibility of the *S2E* models (135) underscore the potential of the prediction of latent MMEs in the near future.

To date, significant effort has been devoted to identifying potential biomarkers and developing new technologies that enable more effective probing of latent MMEs. However, given that a low frequency of proviruses in CD4+ T cells (128, 233) is present, exploring a richer array of features that can directly capture the clinical phenomena of HIV infections remains difficult. *DL* models that automatically extract low-level features and generalize them into higher-level features may offer a promising approach for predicting latent MMEs from limited input from a small clinical cohort. It is important to stress that ML-based models with improved domain-specific learning curves enable a robust prediction with minimum data set sizes of the inputs ranging between 500 and 1,000, which is affordable by the majority of HIV cohort studies (234). Furthermore, *GCNs* (132) (see the previous section, Step 2) have demonstrated strong potential to capture biologically meaningful structures and offer superior performance by reducing overfitting in small-cohort biomedical applications (235). Forthcoming efforts should be made toward the discovery of features that specify the transcriptional program of a provirus (productive replication versus inducible and deep latency), rather than reinforcing or maintaining their transcriptional states, thereby enabling a clear discrepancy across distinct latent MMEs.

As a final remark, cell-type-specific gene expression predictions based solely on primary sequences remain a considerable challenge for the field (13). An increasing number of immune cells that either directly or indirectly interact with HIV infections and elicit various immune responses (including immunological memory) that may influence the configuration of latent MMEs should also be taken into account when building a model. The improvements in computing capacity in this context may enhance predictive accuracy for distinct cell types of the HIV reservoir and HIV reservoir compartmentations.

## CONCLUDING REMARKS AND FUTURE PERSPECTIVES

*DL* models are capable of integrating multi-modal biological data—from HIV IS, reservoir cellular state, the integrity of the HIV genome, HIV transcription, and latency reversal—positioning it uniquely to decode the complex and heterogeneous nature of latent MMEs. As previously mentioned, at present, it remains difficult to precisely define latent MMEs. Most likely, the observation of latent MMEs can be identified separately for each feature or characterized as an ensemble of all features. Therefore, it is crucial to carefully choose or integrate various features before engaging in *DL* modeling, as this can result in differing predictive results. Encouragingly, *DL* models can capture the non-linear interactions between features retrieved from different biological dimensions that collectively define the likelihood of distinct latent MMEs. A correlation between observed phenomena related to HIV/AIDS disease progression and a wide range of features resulting from a variety of “omics” approaches has been unveiled. More efforts of future research should focus on illuminating which features identified from latent MMEs are truly causative versus correlative. In this respect, *DL* models offer an opportunity to retrieve crucial features represented in distinct latent MMEs and lead us to explore more about the nature of latent MMEs from a mechanistic point of view. Apart from the prediction of latent MMEs, when more “omics,” clinical and patient-specific data sets are available for *DL* in the near future, more converging trends, including (i) establishing a comprehensive single-cell atlas of HIV reservoirs at a single-virus level through integrated single-cell “omics” modalities and near-full-length proviral sequencing (37), (ii) tracking the evolutionary trajectory of latent MMEs and those harboring proviruses at a deeper level of latency based on clinical longitudinal data sets, (iii) novel antiretroviral drugs discovery and screening, (iv) AI-based medical decision making for diagnosis and therapies, and (v) constructing digital twins-driven (236, 237) patient-specific models of the HIV reservoir, can be anticipated. Such knowledge is expected to guide us to precise HIV interventions and the development of personalized HIV regimens.

### Glossary

#### Deep learning (DL)

A class of machine-learning approaches capable of identifying highly complex patterns in large datasets (reviewed in reference 135).

#### End-to-end models

Machine learning models that embed the entire data-processing pipeline to transform raw input data into predictions without requiring a preprocessing step (reviewed in reference 137).

#### Deep neural networks

A wide class of machine learning models with a design that is loosely based on biological neural networks (reviewed in reference 137).

#### Convolutional neural networks

A neural network architecture using convolutional layers that extract local patterns at different spatial hierarchies (reviewed in reference 135).

#### Recurrent neural networks

*RNNs* are designed for sequential data processing and are crucial for tasks involving time-series or language data (129).

#### Transformers

A neural network architecture based on the concept of self-attention that weighs the importance of different parts of the input, allowing long-range dependencies to be captured (reviewed in references 130, 131).

#### Graph convolutional networks

Neural networks that process graph-structured data, generalizing the concept of convolution from images to graphs. The same neural network is applied to each node and edge in the graph.

#### Capsule networks

It is designed to enhance model hierarchical relationships and the processing of information by using groups of neurons known as “capsules,” which handle more complex tasks than the typical neurons in *CNNs* (133).

#### Generative adversarial networks

It consists of two interconnected neural networks—the generator and the discriminator—and engages in a continuous game-theoretic competition during training (134).

#### Attention-based deep learning

A deep learning model embeds an additional layer, so-called the *attention layer*. This layer takes the feature vector after *convolution-pooling operations* as input, and then computes a score indicating whether the neural network shall pay attention to the sequence features at that position.

#### Attention mechanisms

A particularly designed layer (i.e., *the attention layer*; see the definition above) to extract important regions of input data by training an additional neural network that learns the relative importance of each input position from local features (231).

#### One-hot coding

A technique for converting categorical data into a numerical format that machine-learning algorithms can process.

#### Position weight matrix

Representation of motifs in aligned DNA sequences as matrices that indicate the importance at each nucleotide position (reviewed in reference [135]).

#### Multilayer perceptron

It is one of the most widely used types of neural networks for supervised learning tasks (143).

#### Bidirectional recurrent neural network

It is a type of *recurrent neural network* architecture that processes sequential data in both forward and backward directions simultaneously, allowing the model to capture context from both past and future states at any given point in a sequence.

#### Sequence-to-expression (S2E) model

This model enables the prediction of gene expression from DNA sequence alone (reviewed in reference 135).

#### Long short-term memory

It is a special type of *RNNs* designed to learn and remember information over long sequences of data (155).

#### Gated recurrent unit

It is a type of *RNNs* and is used to gate mechanisms to selectively update the hidden state at each time step, allowing remembering important information (195).

#### Autoencoder/variational autoencoder

It is an artificial neural network architecture and can be viewed as two coupled, but independently parameterized models—the encoder or recognition model, and the decoder or generative model (150).

#### Graph neural networks

It is a class of *DL* models designed to perform inference on data structured as graphs—that is, data represented by vertices (entities) and edges (correlations between entities).

#### Conditional random fields

It is a type of discriminative probabilistic graphical model used to model the conditional probability of a set of output variables given a set of input variables, particularly well-suited for structured prediction tasks where outputs are interdependent (207).

#### Diffusion models

It is a class of generative *D*L models that learn to generate data, such as images, audio, video, and so on (209).

#### Message passing NN

It is a general and unified framework for *GNNs* that formalizes how information is propagated and aggregated across graph structures (215).

#### Neural ODE

It is a class of *DL* models that parameterizes the derivative of a hidden state using a neural network, rather than defining discrete layer-by-layer transformations (218).

#### The convolution layer

A neural network layer that processes data stored in *n*-dimensional arrays, such as images. When applied to DNA sequences, a convolutional layer can be interpreted as a set of *position weight matrices* (see the definition above) scanned across the sequence (reviewed in reference [137]).

#### The pooling layer

This layer aggregates the activations in contiguous bins across the positional axis, typically taking the maximal or average activation for each *channel*—an axis other than one of the positional axes (reviewed in reference 137).

#### The fully connected layer

A neural network layer, in which every input contributes to the computation of every output (reviewed in reference 135).

#### Kernel

A filter, which is a small matrix that can detect specific patterns in the input sequence through the process of convolution (reviewed in reference 135).
